# Prevalence of class 1 integron in *Escherichia coli* isolated from animal sources in Iran: a systematic review and meta-analysis

**DOI:** 10.1186/s41182-020-00202-1

**Published:** 2020-04-07

**Authors:** Maryam Karimi Dehkordi, Mehrdad Halaji, Samereh Nouri

**Affiliations:** 1 Department of Clinical Sciences, Faculty of Veterinary Medicine, Shahrekord Branch, Islamic Azad University, Shahrekord, Iran; 2 Department of Microbiology, School of Medicine, Isfahan University of Medical Sciences, Isfahan, Iran; 3 Department of Microbiology, Clinical Laboratory of Al Zahra Medical Centre, Isfahan University of Medical Sciences, Isfahan, Iran

**Keywords:** *Escherichia coli*, Antibiotic resistance, Integron, Meta-analysis, Animal source

## Abstract

**Background:**

Among the genetic elements, integrons may contribute to the widespread incidence and spreading of antibiotic resistance among *Escherichia coli* isolates. Accordingly, this review aims to investigate the prevalence of class 1 integron in *E. coli* isolated from animal sources in Iran.

**Methods:**

This systematic literature search was performed from January 1, 2000 to the end of May 1, 2019.

Then, publications that met our inclusion criteria were selected for data extraction and analysis. Also, the quality of included studies was independently assessed by two researchers based on the Joanna Briggs Institute. Meta-analysis was performed by the Comprehensive Meta-Analysis (CMA) software using the random effects model, Cochran’s Q, and *I*^2^ tests. Publication bias was estimated by funnel plot and Egger’s linear regression test.

**Results:**

Based on inclusion criteria, five studies were included to meta-analysis. From those studies, the pooled prevalence of integrons was 33% (95% CI, 23.8–43.7%) ranging from 23.8 to 52.4%. There was a significant heterogeneity among the 5 studies (*χ*^2^ = 11.73; *p* < 0.019; *I*^2^ = 65.91%). Additionally, Begg’s and Egger’s tests were performed to quantitatively evaluate the publication biases. According to the results of Begg’s test (*z* = 1.22, *p* = 0.22) and Egger’s test (*t* = 3.03, *p* = 0.056), a significant publication bias was not observed.

**Conclusions:**

Our finding revealed the relatively high prevalence of class 1 integrons among E. coli isolates. Moreover, there was a significant heterogeneity among studies and subgroup analysis also showed that there was no difference about prevalence of class 1 integrons among different sample source.

## Background

Enterobacteriaceae is a diverse group of Gram-Negative Bacteria (GNB) causing various diseases in humans and animals. In this family, *Escherichia coli* (*E. coli*) is the most common commensal bacteria in the gastrointestinal tract microflora of both humans and animals [1, 2]. As carriers of the bacteria, *E. coli* can be found in animals such as cattle, sheep, pigs, deer, dogs, and poultry [3]. Infected animals can shed the bacteria in their feces and easily contaminate their environment. People in contact with infected animals and their environment are exposed to these bacteria [4]. Antibiotics are commonly used for prophylaxis and treatment of infected humans and animals. Inadequate selection and abuse of antibiotics may lead to resistance in various bacteria and make the treatment of bacterial infections more difficult [5, 6]. Multidrug-resistant (MDR) in GNB, especially in *E. coli*, has become one of the major challenges to human and animal health [5, 7]. Intrinsic and acquired resistance mechanisms are the cause of antimicrobial resistance (AMR) among *E. coli*. *E. coli* strains have a considerable capacity to acquire resistance genes mostly through horizontal gene transfer. Horizontal gene transfer is an important mechanism for the rapid spread of antibiotic resistance genes between GNB species [8, 9].

In recent years, different acquired resistance mechanisms, including bacteriophages, transposons, plasmids, and integrons, have been identified involving the spread of resistance genes in the bacteria [10]. Among the genetic elements, integrons may also contribute to the widespread incidence and spread of antibiotic resistance [11]. These elements are capable of capturing, integrating, and mobilizing antibiotic resistant gene cassettes. The integrons were classified into three important classes, including 1, 2, and 3, based on the genetic relatedness of the integrase *intI* gene sequence. Among them, class 1 integrons are more frequent in GNB [12].

Metallo-beta-lactamase (MBL) is commonly encoded on the gene cassettes harboring class 1 integron and circulated simply in bacterial populations [13]. Additionally, newly extended spectrum beta lactamases (ESBL)-encoding genes are usually located on integron-like structures (such as *blaCTX*-*M*, *blaGES*, or *blaVEB*-*1*) [14]. In this regard, since antibiotics are widely used in animal sources, the presence of integrons and antibiotic resistant gene in animal products has become a widespread concern [15].

Thus, understanding the prevalence of class 1 integrons among animal sources can play a crucial role in controlling and transferring them to humans, which may help to prevent the spread of resistance determinants in GNB. There are significant data gaps regarding class 1 integron from an animal source in Iran. Accordingly, this review aims to investigate the prevalence of class 1 integron in *E. coli* isolated from animal sources in Iran.

## Methods

### Search strategies

The present study was designed according to the Preferred Reporting Items for Systematic Reviews and Meta-Analyses (PRISMA) guidelines (Additional file 1). A systematic literature search was done from January 1, 2000 to the end of May 1, 2019 (using the Web of Science, PubMed, Scopus, and Google Scholar electronic databases) to search for studies published by Iranian authors. The keywords search was carried out using medical subject headings (MeSH) terms such as “*Escherichia coli*,” “*E. coli*,” “Integron,” AND “Int1” “antibiotic resistance,” “incidence,” “distribution,” “prevalence,” and related terms AND “Iran” individually or combined together in the title or abstract keywords fields.

### Selection criteria and quality assessment

The search was limited to original articles published in English or Persian language with English abstract indexed in the Web of Science, PubMed, Scopus, and Google Scholar databases.

The inclusion criteria was as followed: The cross-sectional studies reporting the frequency and prevalence of integron in *E. coli* isolates obtained from animal sources and articles published in English or Persian language with English abstract. In studies that samples were obtained from sources other than animal, and the primary sample size unclear and the review, case reports and letters to the editor’s articles were excluded.

Two reviewers determined whether the articles met the inclusion criteria, the titles, abstracts, and full texts of articles independently screened with the related keywords in the databases. Discrepancies among reviewers were resolved by consensus. Furthermore, searching references from included studies/reviews as manual search was performed.

### Quality assessment and data extraction

The quality of included studies was independently assessed by two researchers based on the Joanna Briggs Institute and any disagreements were resolved by consensus.

We collected detailed information by the researchers about the first author’s name, the year of publication, the source of samples, the source of isolation, sample size, and the frequency of class 1 integron.

### Statistical analysis

Analysis of data was done using the Comprehensive Meta-Analysis Software Version 2.2 (Biostat Company). The random-effects model was used to estimate the pooled prevalence and corresponding 95% confidence interval (CI). Statistical heterogeneity between studies was estimated with the Cochran’s Q statistic and I-square (*I*^2^) test. The funnel plot, Begg’s rank correlation test, and Egger’s weighted regression tests were used to evaluate the possibility of publication bias (*p* < 0.05 was considered as indicative of statistically significant publication bias).

Possible sources of heterogeneity were calculated using sensitivity analysis and subgroup analysis based on the location of the study, and sample source was assessed to calculate possible sources of heterogeneity

## Results

### Database search and characterization of studies

**Fig. 1:**
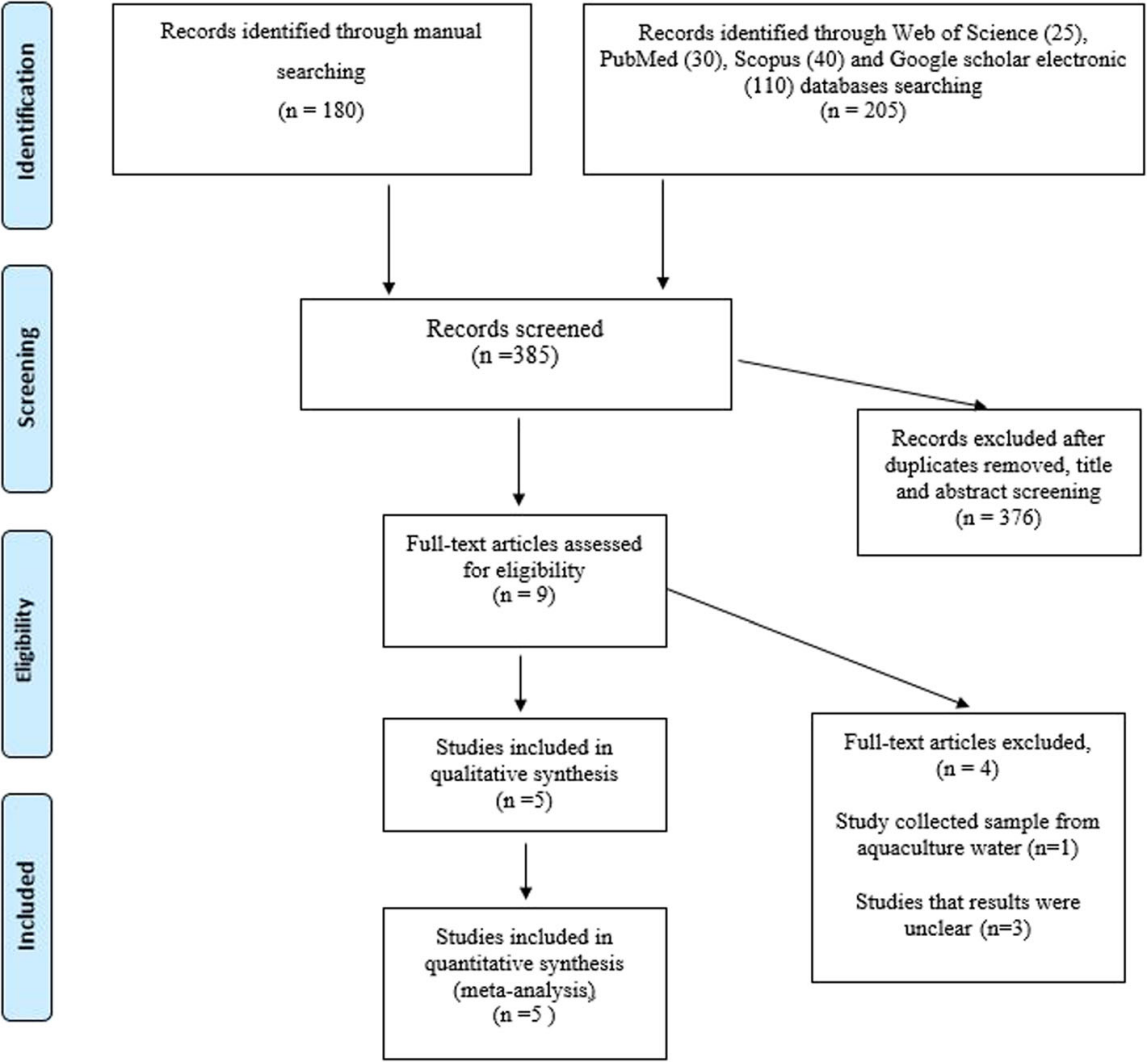
Flow chart of study selection for inclusion in the systematic review

The database search yielded 205 citations. Among them, 196 were removed by index, title, and abstract screening and 9 were retrieved in full text [16–24]. Of 9 reviewed studies, in a study, samples were collected from aquaculture water [16], and the results of three studies were unclear thus, four studies were excluded upon a full-text search [22–24]. Finally, based on inclusion criteria, 5 studies were included to meta-analysis [17–21]. The searching process for collection of qualified studies is shown in Fig. 1. The full results of the selected articles such as sample size, type of animal, the frequency of *Int1*, and source of samples are presented in Table 1. All studies examined the frequency of *Int1* in cattle, goats, chicken, sheep, and calves. Also, all of isolates were recovered from faecal sample.

**Table 1:** Characteristics of studies included in the meta-analysis

Study	Publication year	City or province	Sample source	Type of animal	Sample size	Int1	Diagnostic method	Ref
Taghadosi	2019	Kerman	Faecal	Healthy cattles and goats	18	9	PCR	[17]
Staji	2018	Tehran	Diarrheic	Cattle and poultry farms	63	15	PCR	[18]
Kohansal	2018	Fasa	Stool samples	Diarrheic calves	71	23	PCR	[19]
Kheiri	2016	Karaj	Faecal	Healthy chicken, cattle, and sheep	150	36	PCR	[20]
Bakhshi	2014	Tehran	Diarrheic	Calves	21	11	PCR	[21]

### Prevalence of class 1 integrons

**Fig. 2:**
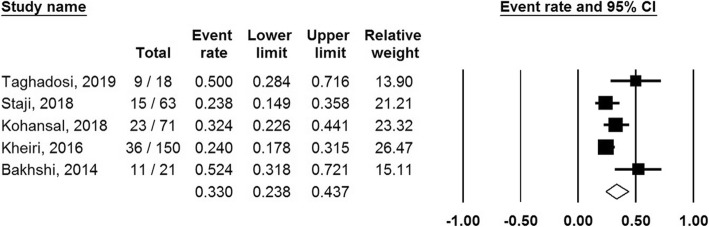
Forest plot of the meta-analysis of *integron* in animal sources

**Fig. 3:**
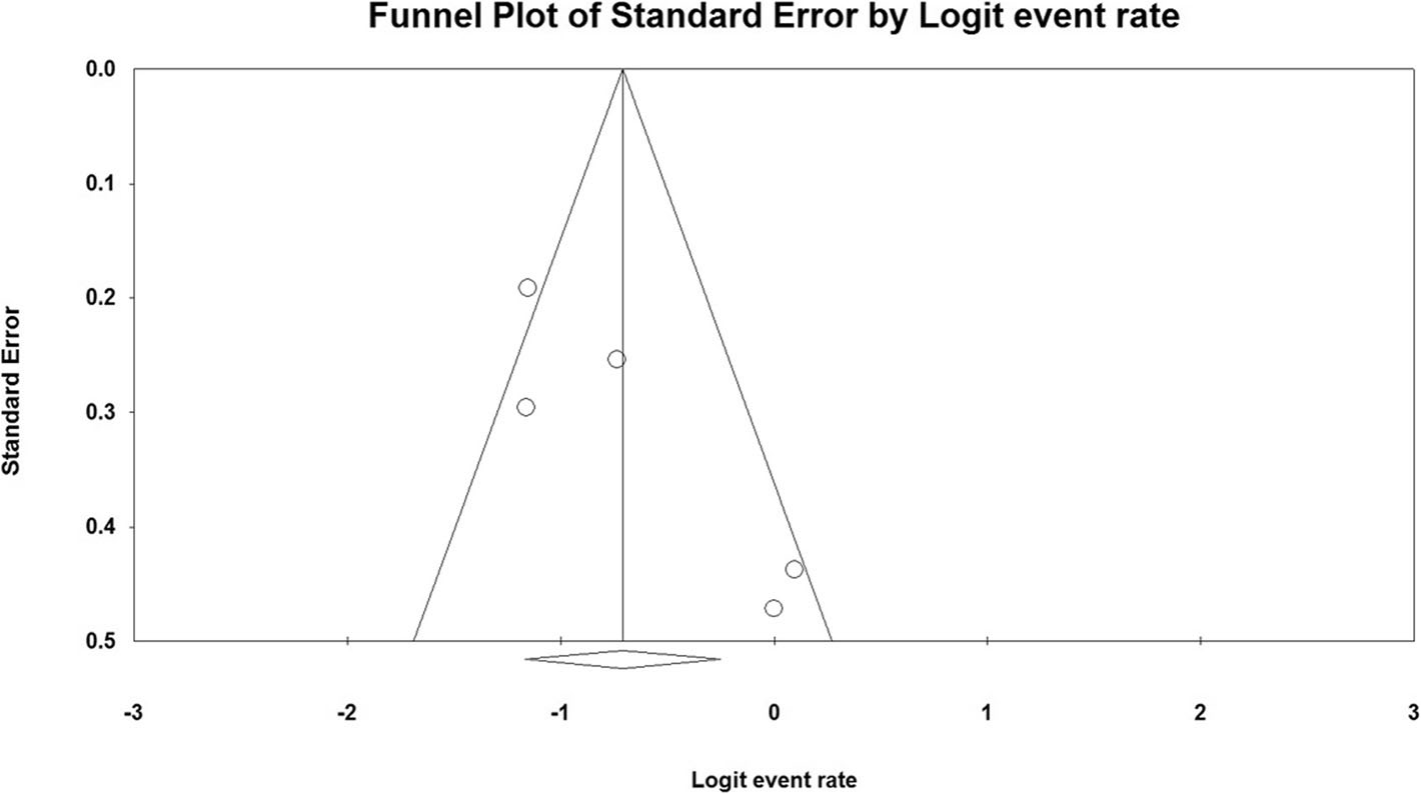
Funnel plot of publication bias for the included studies

From included studies, the pooled prevalence of class 1 integron was 33% (95% CI, 23.8–43.7%) ranging from 23.8 to 52.4% (Fig. 2). There was a significant heterogeneity among the 5 studies (*χ*^2^ = 11.73; *p* < 0.019; *I*^2^ = 65.91%). Additionally, the funnel plot, Begg’s, and Egger’s tests were performed to quantitatively evaluate the publication biases. The funnel plot showed no evidence of asymmetry. According to the results of Begg’s test (*z* = 1.22, *p* = 0.22) and Egger’s test (*t* = 3.03, *p* = 0.056), a significant publication bias was not observed (Fig. 3).

### Subgroup analysis of prevalence of class 1 integrons

**Fig. 4:**
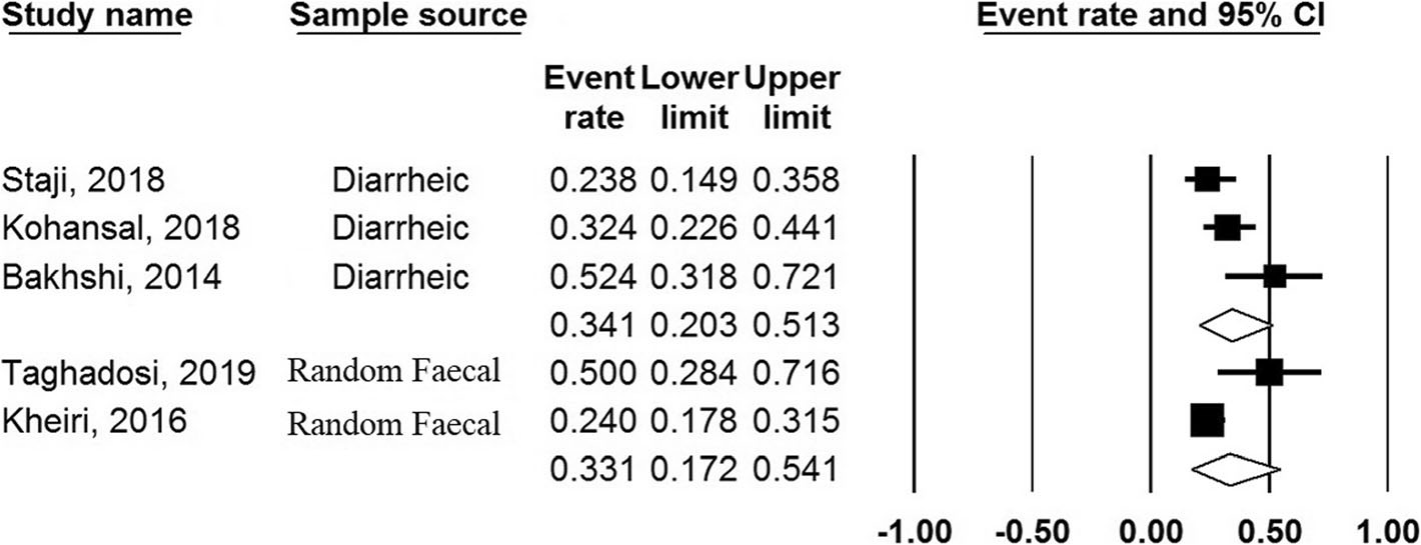
Forest plots of the prevalence of class 1 integron based on sample source

**Fig. 5:**
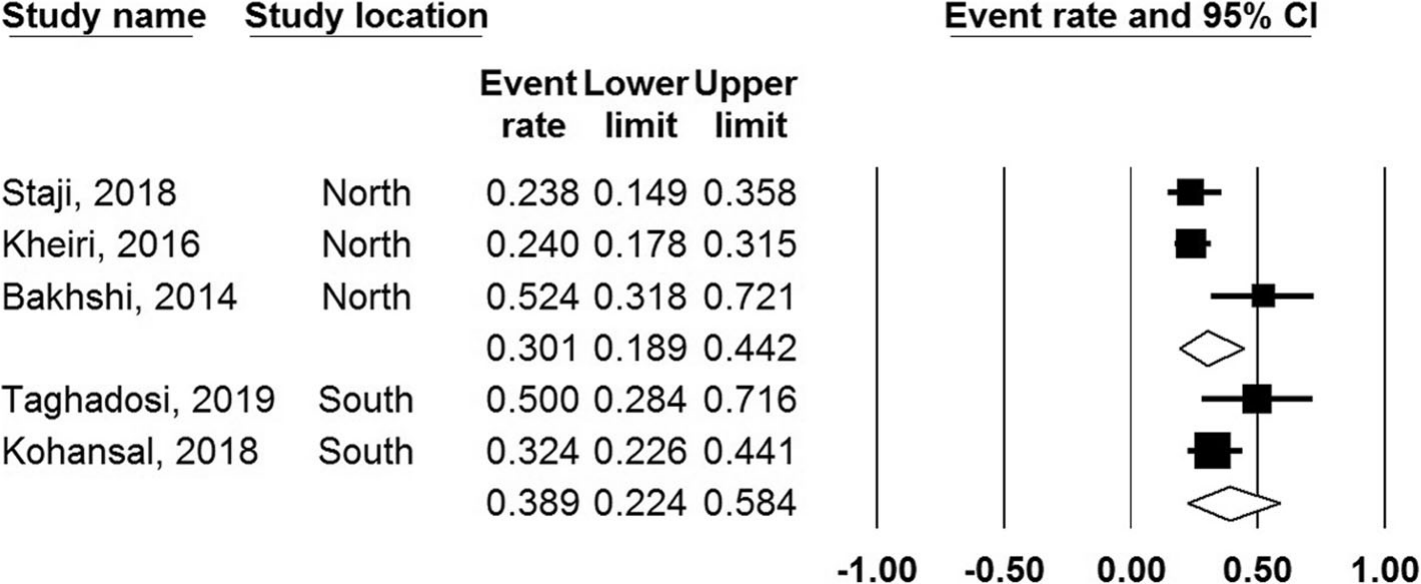
Forest plots of the prevalence of class 1 integron based on region

The finding of subgroup analysis based on sample source revealed that the prevalence of class 1 integrons among diarrheic and healthy samples were 34.1% (95% CI, 20–51%) and 33.1% (95% CI 17–54%), respectively. Moreover, subgroup analysis based on region showed that the prevalence of class 1 integrons were 30% and 39% in the North and South of the Iran, respectively (Figs. 4 and 5).

### Sensitivity analysis

**Fig. 6:**
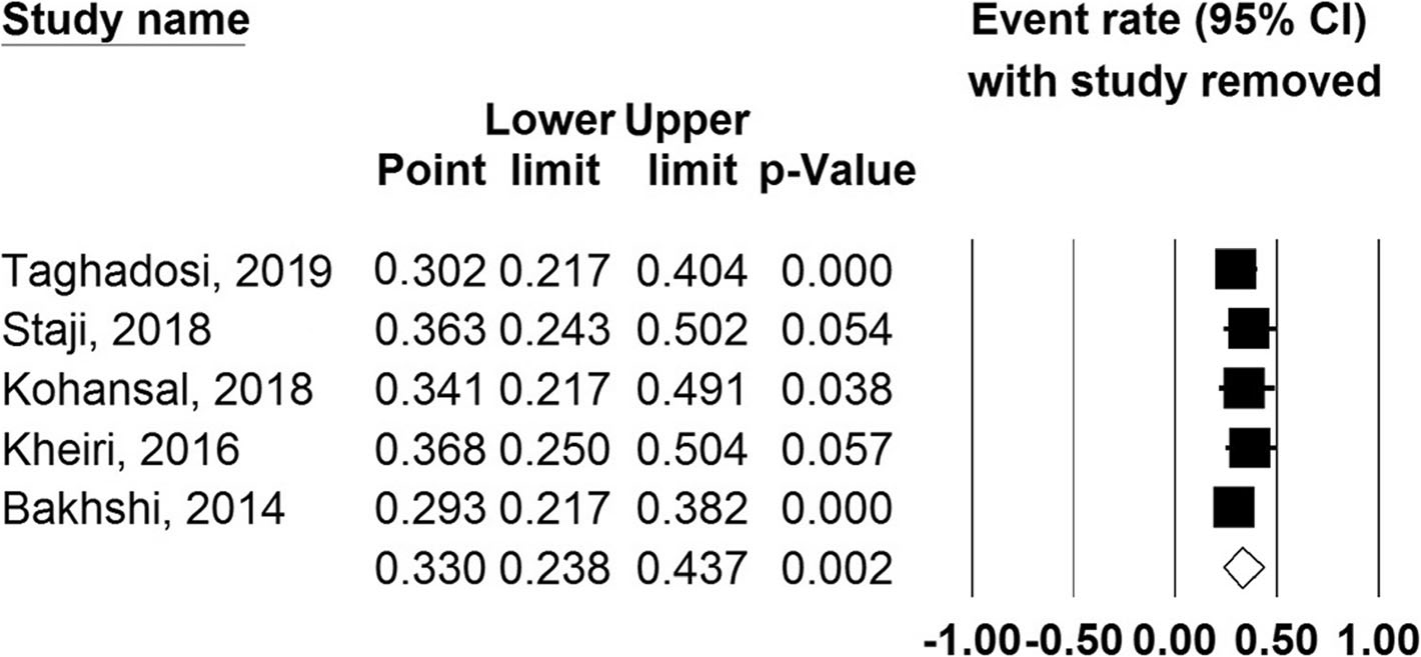
Sensitivity plot of eligible studies included in the meta-analysis

According to sensitivity analysis results, none of the eligible studies has the ability to change the overall prevalence substantially (Fig. 6).

## Discussion

Nowadays, there are considerable data suggesting that excessive and improper administration of drugs to farm animals without being aware of the consequences of them leads to increasing the antibiotic-resistant bacteria, especially in humans [25–28]. Unfortunately, in Iran, similar to other countries, antibiotic precipitation in farm animals is relatively frequent and widespread [29, 30]. Therefore, owing to the importance of drug resistance in animal farms and the possibility of their transmission into clinically important bacteria, the evaluation of drug resistance, especially the antibiotic resistance mechanisms, should be considered [31]. Integrons and other related gene cassettes are regarded as important genetic determinants of MDR strain in *E. coli.* These elements participate particularly in the horizontal transmission of genes involved in antibiotic resistance among clinical isolates of *E. coli* [17, 20].

To the best of our knowledge, this is the first comprehensive meta-analysis addressing the frequency of class 1 integrons in *E. coli* obtained from a different animal in Iran. Based on this meta-analysis, the frequency of class 1 integrons in *E. coli* recovered from domestic animal varied from 23.8 to 52.4%. Furthermore, the pooled prevalence of class 1 integrons was calculated as 33%. The prevalence of class 1 integrons in *E. coli* isolates has been variable in different reports ranging from > 50% in studies conducted in Chile (37.3% in Poultry), Korea (31.7% in poultry and Swine), China (41.94% in chicken farm), China (36.8% in bovine), and Norwegian origin (12% in swine and poultry meat), being partially consistent with our findings [15, 32–35]. Compared to our study, Su et al. investigated the occurrence of integrons in Enterobacteriaceae isolated from integrated fish farms. The *intI1* gene was found in 83.7% isolates of Enterobacteriaceae, which had a higher rate than ours [36].

The low frequency of class 1 integrons may be owing to presence of other resistance mechanisms such as intrinsic resistance factors, reduced permeability of the bacterial outer membrane, production of beta-lactamase enzymes, and overexpression of efflux pumps [37–39]. Furthermore, the discrepancy in the prevalence of class 1 integrons in different studies can partially be owing to geographical variation, type of studies animals, and improper and excessive use of antibiotic [17, 20]. In one study conducted by Zeeshan Khan et al., class 1 integrons in multidrug-resistant uropathogenic *E. coli* isolates were assessed. Based on their results, 79% of MDR *E. coli* isolates harbored class 1 integrons [38]. Moreover, in Iran, in one study conducted by Pormohammad et al., the prevalence of integron classes in Gram-negative clinical isolates was reported. Based on this report, the prevalence of integron class 1 was 41%, being inconsistent with the rate of integron class 1 in E. coli isolated from animal sources [40]. According to these literature reviews, the higher rate of integrons can lead to significant antibiotic resistance and consequently the emergence of ESBL and MDR isolates, which could be a serious risk to healthcare systems as well as livestock and poultry industries [41, 42]. The frequency of class 1 integron in Iran is comparable to human resources. Although compared to human samples, the overall frequency of class 1 integron in animal resources was low, awareness about this information is vital owing to a higher occurrence of antibiotic resistance in these isolates [24, 41].

The role of class 1 integron in resistance to new generation antibiotics was characterized. Thus, based on the structure of class 1 integron gene cassettes, the presence of ESBLs and Metallo-β-lactamases (MBLs) hydrolyzing third and fourth generation cephalosporins and carbapenems leads to resistance to these classes of antibiotics [43, 44]. Integrons, as mobile elements, can transmit and carrier the resistance genes from one organism to others; this challenge in livestock and poultry industries is extremely important, since the performance of infection control programs is poor [45–47]. According to our analysis, significant publication bias was not observed. However, it seems that various factors have stronger effects on the frequency of class 1 integron. Source of samples, geographical distribution, and sample size are among the factors in variation pathotypes of *E. coli*. Nevertheless, the analysis revealed that the weight of studies could not be reflected as an influence factor, and exclusion of any study has no remarkable effect on the estimated pooled prevalence. The main limitation of our study was heterogeneity among the included studies; thus, the results should be interpreted with caution.

## Conclusions

Our findings demonstrated the relatively high prevalence of class 1 integrons among E. coli isolates. Furthermore, there was a significant heterogeneity among the studies. Moreover, subgroup analysis showed no difference in the prevalence of class 1 integrons among different sample sources.

## Supplementary information

**Additional file 1.** Study design according to the Preferred Reporting Items for Systematic Reviews and Meta-Analyses (PRISMA) guidelines.

## Data Availability

The datasets used and/or analyzed during the current study are available from the corresponding author on reasonable request

## Abbreviations

AMR: Antimicrobial resistance
CI: Confidence interval
*E*. *coli*: *Escherichia coli*
GNB: Gram-Negative Bacteria
PRISMA: Preferred Reporting Items for Systematic Reviews and Meta-Analyses
MDR: Multidrug-resistant
MeSH: Medical subject headings
